# A Hybrid CFD Platform for Colloidal Fouling Prediction in Electrodialysis

**DOI:** 10.3390/membranes15120375

**Published:** 2025-12-06

**Authors:** Francesco Volpe, Giuseppe Battaglia, Andrea Cipollina, Giorgio Micale, Alessandro Tamburini

**Affiliations:** Dipartimento di Ingegneria, Università degli Studi di Palermo, Viale delle Scienze, 90128 Palermo, Italy; francesco.volpe03@unipa.it (F.V.); andrea.cipollina@unipa.it (A.C.); giorgiod.maria.micale@unipa.it (G.M.); alessandro.tamburini@unipa.it (A.T.)

**Keywords:** electromembrane processes, computational fluid dynamics, fouling, ion exchange membranes, concentration polarization, 3-D model

## Abstract

Fouling phenomena are among the main issues in membrane processes, worsening unit performance and membrane properties. So far, few modelling approaches have been proposed to predict colloidal fouling in electromembrane-based technologies. This work presents an original simulation platform that couples computational fluid dynamics (CFD) simulations with electrodialysis (ED) and colloidal fouling models to investigate the impact of colloidal deposition at the channel and unit scales of ED systems. Fluid dynamics, salt transport and fouling layer growth were all addressed. The model was calibrated and validated with colloidal fouling data from the literature. The regions more susceptible to fouling growth were identified. Polarization phenomena, as well as the increase in pressure losses and electrical resistance over time, were evaluated.

## 1. Introduction

Today, much of the effort in technological innovation is oriented toward the advancement and optimization of sustainable processes. Sustainability and circular economy approaches are gaining increasing prominence, reflecting the necessity of shifting away from a fossil fuel-based economy and moving beyond conventional production models. In this framework, membrane separation processes represent key technologies that can be widely implemented to conduct conventional operations with enhanced efficiency and selectivity [1,2].

A notable example is electrodialysis (ED), a well-established technology that enables the selective separation of charged species [3]. ED technology has traditionally been employed for the desalination of brackish water [4]—where it stands as a competitive alternative to reverse osmosis [5,6]—and has more recently been applied to a broad range of emerging sectors, such as biorefinery, the food industry, nutrient recovery, and wastewater treatment [7,8,9,10,11,12].

ED technology belongs to the class of ion-exchange membrane (IEM) processes, in which polymeric membranes incorporating charged functional groups are utilized to achieve the selective transport of ions based on their electrical charge. In an ED unit, cation-exchange membranes (CEMs) and anion-exchange membranes (AEMs) are stacked alternately with polymeric spacers placed in between to create flow channels for the solutions [13]. The pile of membranes and spacers is confined between two electrodes located at opposite ends of the unit. When an electrical potential difference is applied between the electrodes, ion migration between channels is induced. The selective migration of ions through channels creates concentrated and dilute streams [14].

As in most membrane-based processes, fouling represents a major challenge in electrodialysis, particularly when the feed solution contains complex mixtures of dissolved species [15]. Fouling can be described as “the undesirable deposition of living organisms or non-living substances on membranes” [16]. This phenomenon leads to detrimental effects on system performance, such as (i) increased pressure losses, (ii) a reduced membrane permselectivity and (iii) a higher membrane and unit electrical resistance [17,18,19].

Fouling phenomena can occur in various forms, such as biofouling, organic fouling, scaling and colloidal fouling, depending on the concentration, composition and purity of the dissolved or suspended species [16].

Specifically, *colloidal fouling* occurs when small, suspended charged particles, known as *colloids*, attach to membrane surfaces due to electrostatic forces. Two pioneering studies on colloidal fouling in ED were conducted by Grossman et al. [20] and Korngold et al. [21] in the 1970s. Grossman et al. [20] developed a mathematical model to describe the reduction in the limiting current density due to the formation of a fouling layer consisting of both neutral and colloidal matter. The work found that charged fouling layers exerted a more pronounced effect than neutral fouling layers. Moreover, the study assessed that fouling depended not only on the composition of foulant species, but also on hydrodynamic conditions and salt concentration. Korngold et al. [21] reported a predominant tendency of AEMs to be fouled by charged substances in ED tests with sodium humate. The authors highlighted that operating conditions promoting polarization phenomena, such as low flow velocity, low salt concentration and high current density, exacerbated the extent of fouling. Fouling was found to be more severe on rough membranes than on smooth ones. The authors also observed that alkaline washing, conducted with a reversed electric current, was successful for stack regeneration.

Fouling on CEMs was investigated by Sosa Fernandez et al. [22], who studied the case of ED for the desalination of polymer-flooding-produced water. The performance of an ED unit fed with several feed solutions, where NaCl, partially hydrolyzed polyacrylamide (HPAM), divalent cations such as Ca^2+^ and Mg^2+^, and traces of crude oil were dissolved, was evaluated to assess fouling caused by HPAM. Membrane characterization after the tests, performed with scanning electron microscopy (SEM) and energy-dispersive X-ray spectroscopy (EDX), Raman spectroscopy and contact angle measurements, provided an in-depth assessment of the membrane deterioration caused by fouling and the impact of each dissolved ion species. The study also examined the reversibility of fouling, highlighting the need for predictive models for process control and the planning of cleaning operations.

Several studies can be found in the literature on the modelling of the ED process, exploring different approaches from simplified 1-D models [13,23,24] to 2-D formulations [25,26,27,28] to more complex 3-D ones [29,30,31]. In this latter case, mass transport and fluid dynamics are accurately resolved at the low scale of spacer-filled channels. Several studies also investigated electro-osmosis and electrophoresis phenomena through 3-D CFD analyses [32,33,34,35,36], where mixing and polarization phenomena at the boundary layer were thoroughly addressed. However, neither of these studies nor the aforementioned ED models embedded a description of colloidal fouling phenomena, which are mostly analysed in other membrane-based technologies. Liu et al. [37] proposed a colloidal fouling model for reverse osmosis (RO) and nanofiltration (NF). The model was based on XDLVO theory, introducing a collision–attachment approach describing fouling deposition on membranes. Although the influence of fluid dynamics was not fully accounted for, the model results were in good agreement with experimental data, successfully capturing trends of water flux decline. Some years later, the same authors [38] incorporated Monte Carlo simulations into the collision–attachment approach. The model described the interactions between foulants and clean or fouled membranes. Other approaches have been proposed by Wang et al. [39] for the study of the colloidal fouling for surface filtration processes with ceramic membranes and by Lohaus et al. [40] within the context of microfiltration.

Within the ED context, De Jaegher et al. [41] developed a neural differential equations-based approach to predict colloidal fouling behaviour caused by humic acid under several operating conditions. A sensitivity analysis based on the Sobol method was performed to assess the relative importance of the input variables (electric current, flow velocity and salt concentration) on fouling rate. The authors highlighted that flow velocity was the most relevant parameter. The model predicted both linear and exponential fouling regimes, accurately describing the evolution of stack resistance due to fouling.

The same authors [42] developed a hybrid model combining a 2-D description of the ED process, which did not explicitly account for fouling, with mechanistic colloidal fouling equations, and a neural network approach to estimate the growth probability of colloids and relate it to the electrical resistance and the current density of an ED system. Numerical stack resistance values predicted using the hybrid model agreed very well with experimental data from the literature [43].

This brief literature review highlights the limitations of the proposed modelling approaches addressing fouling in ED units. The CFD models presented so far have only modelled the flow field and mass transfer for clean channels, not accounting for fouling phenomena. Other modelling approaches developed to predict fouling deposition, although capable of forecasting the evolution of ED performance, did not incorporate a physical framework to describe fouling or its effects on fluid dynamics and mass transport within the channels (see data-driven models). The present work introduces a novel comprehensive numerical tool aiming at providing an in-depth description of the colloidal fouling in ED units at both channel and unit scales. Specifically, this is the first model that couples computational fluid dynamics (CFD) simulations with a colloidal fouling model comprising physically meaningful equations. This hybrid framework accurately depicts fluid flow and mass transfer in ED channels with spacers, also accounting for an integrated fouling layer. The simulation platform enabled an accurate investigation of fouling effects at both macroscopic and microscopic scales, capturing its impact on pumping power as well as the three-dimensional concentration distribution, thereby highlighting the polarization phenomena.

## 2. Modelling Approach

Mass transport and colloidal fouling in an ED spacer-filled channel were analysed by coupling a 3-D CFD approach with a fouling model. The hybrid model couples 3-D CFD simulations to describe the fluid flow and salt transport in the channel, both in clean and fouled conditions, while fouling was described by a dedicated 3-D model, whose resolution was made by an in-house script that solved mechanistic equations for colloid deposition.

### 2.1. CFD Model

A 3-D CFD model was developed following the approach presented in Gurreri et al. [29]. The fluid flow velocity and trajectories in a spacer-filled channel, as well as the influence of electric current on the salt concentration distribution, were simulated.

**Figure 1:**
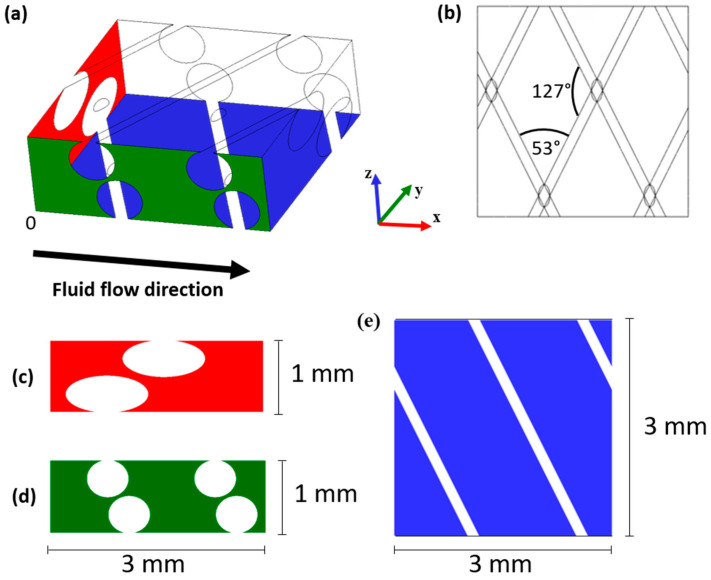
(**a**) The geometry of the computational volume. (**b**) The wireframe from a view perpendicular to the z-axis. Wall sections perpendicular to the (**c**) x-axis, (**d**) y-axis and (**e**) z-axis. The upper and lower walls (the two walls that have the z-axis as their normal) are the membranes. Specifically, the wall located at z = 0 is the AEM, whereas the one located at z = 1 mm is the CEM.

Figure 1 provides a picture of the simulated spacer-filled fluid channel.

The main computational domain, Figure 1a, consists of a rectangular spring, representing a periodic unit of a channel section, where the areas corresponding to the spacer filaments were excavated. The rectangular spring has dimensions of 3 mm × 3 mm × 1 mm along the x-, y- and z-axes, respectively, corresponding to two repetitive units of spacer meshes. The spacer filaments have a diameter of 0.5 mm, and each one is in contact with one of the two membranes. The angles formed between the filaments are equal to 53° and 127°, while the pitch between filaments in contact with the same membrane is 1.5 mm; see Figure 1b. Since spacer geometry and flow velocity were not reported in the literature data used to validate the model, a spacer configuration (channel thickness and filament geometry) and a flow velocity representative of typical ED unit operation [3] were selected for the CFD model.

An incompressible Newtonian fluid under stationary regime was assumed for the CFD simulations. These steady-state conditions were justified by the low Reynolds numbers encountered in the clean channel. The steady-state Navier–Stokes and continuity equations describe the fluid flow inside the ED channel:

(1) $$\rho \frac{\partial u}{\partial t}+\rho u\nabla \cdot u=-\nabla p+\mu \nabla^{2}u$$

(2) ∇·u=0

where **u** is the velocity vector, p is the pressure, μ is the dynamic viscosity and ρ is the density of the fluid. Given the low concentration of dissolved species, the properties of pure water were assumed for viscosity and density, being 8.9 × $10^{-4}$ Pa∙s and 997 kg/m^3^, respectively. Laminar steady-state simulations were conducted, as preliminary unsteady calculations confirmed that the flow remained essentially stationary. The salt transport resolution was based on the approach proposed by Newman et al. [44]. Under the assumptions of (i) a binary electrolyte and (ii) local electroneutrality, and from the considerations reported in Gurreri et al. [29], the final equation solved to describe salt transport was as follows:

(3) $$\nabla \cdot \left(Cu_{0}\right)=\nabla \cdot \left[D\nabla C\right]$$

where *C* is the salt concentration, **u_0_** is the solvent velocity vector and *D* is the diffusion coefficient of NaCl.

The effect of current on salt transport was appropriately accounted for within the diffusive terms. Specifically, at both membrane–solution interfaces, a salt flux proportional to the current density and transport number was imposed as follows:

(4) $$J=\frac{t^{0}}{z_{i}v_{i}F}i$$

where *z_i_* is the valence (±1 for NaCl), *F* is the Faraday constant (96,485 C/mol), ***i*** is the current density (A/m^2^) and *v_i_* is the stoichiometric coefficient of ionic species *i* (±1 for NaCl).

The flux can be either positive or negative, depending on the channel under study: negative for the dilute channel and positive for the concentrated channel. Since NaCl solutions were considered in this investigation and the transport number was 0.5 for both Na^+^ and Cl^−^, Equation (4) was practically computed as follows:

(5) $$J=\pm \frac{0.5}{F}i$$

#### 2.1.1. Periodicity and Unit Cell Approach

A complete and accurate description of the flow field and scalar transport within a complex geometry, such as an ED channel featuring a spacer, requires a sufficiently fine computational grid. Grids comprising millions of cells have been adopted to simulate a domain of only a few millimetres [29,45,46]. However, simulating an entire ED channel is generally unfeasible, as it would require hundreds of millions of cells. The Unit Cell approach was therefore adopted [47,48]. This approach consists of studying not the whole channel volume, but only a smaller representative domain, typically corresponding to one or a few meshes of the spacers. The Unit Cell is adequate to describe key phenomena, including pressure variations along the channel, scalar distribution and concentration polarization, thereby substantially lowering computational requirements. Since the Unit Cell corresponds to only a section of the whole channel, periodic boundary conditions are applied to the walls located at the edges of the computational domain, representing inlets and outlets of the solution. As reported in Gurreri et al. [29], for fluid dynamics, a momentum source term, S_p_, is added into Equation (1):

(6) $$S_{p}=K_{p}k$$

where *K_p_* is the pressure gradient along the main flow direction, and ***k*** is the main direction vector. Similarly, a concentration source term, *S_c_*, is introduced into Equation (3):

(7) $$S_{C}={-K}_{C}u_{main}=-\frac{JA}{V}\frac{u_{main}}{u_{main\;ave}}$$

where *A* is the membrane surface area in a Unit Cell, *V* is the volume of the simulated domain, $u_{main}$ is the velocity component along the main flow direction (i.e., here, the x-axis) and $u_{main\;ave}$ is its average value. $\frac{JA}{V}$ represents the average value of the source term, while $\frac{u_{main}}{u_{main\;ave}}$ is a local correction term.

Note that this approach can be used to model either the dilute or the concentrate channel of an ED unit, and only the sign of the salt flux and, in turn, the concentration source term would discriminate between compartments. In this work, we focused on the dilute channel, since the literature data used to validate the model refer to experiments in which fouling occurred in the dilute channel, as discussed in Section 2.4.

A full derivation of the equations for the Unit Cell approach can be found in Gurreri et al. [29].

The adoption of CFD simulations enabled the characterization of polarization phenomena, which are central in the analysis of fouling effects. Numerically, the polarization coefficient for the dilute channel can be defined as follows:

(8) $$\theta =\frac{\bar{C_{w}}}{\hat{C_{b}}}$$

where $\bar{C_{w}}$ is the area-averaged concentration at the membrane interface and $\hat{C_{b}}$ is the bulk concentration, which was calculated here as the mass flow-weighted average of the concentration over a cross-section of the Unit Cell (i.e., a y–z plane). From Equation (8), θ is always lower than 1. The closer the θ value is to 1, the less significant the polarization effects. Note that, in a clean channel without a fouling layer, the average concentration at the membrane interface is equal at the two membranes, whereas it may differ when fouling occurs, due to the unequal deposition on the two membranes.

#### 2.1.2. Numerical Details

The commercial finite volume code Ansys^®^ CFX 23 was used to discretize and solve the governing equations. A high-resolution scheme was selected for the treatment of the convective terms. The pressure–velocity coupling was addressed by means of a coupled algorithm. The steady-state simulations were run in double precision and carried out until full convergence was attained. The convergence was considered achieved here for stabilized root mean squared (RMS) residual values lower than 10^−10^.

Both membranes were set as impermeable walls with a no-slip boundary condition for the fluid dynamics. The electrolyte flux was implemented as previously discussed in Equation (5). Spacer filaments were treated as impermeable walls, with zero-flux conditions imposed for the concentration field. Side walls were modelled as periodic boundaries, with opposite walls connected via a translational periodicity condition and a conservative interface flux enforced for momentum and salt transport. Zero velocity and a homogeneous NaCl concentration, equal to that of the experimental cases simulated, were used as the initial guess. A computational grid composed of 1.3 M volumes was adopted as the best trade-off between computational effort and numerical accuracy; see the grid-dependence study reported in Appendix A.2. A pressure gradient was imposed along the main flow direction, namely the x-axis, dictating the solution velocity inside the channel.

### 2.2. Colloidal Fouling Model

The time-dependent colloidal fouling deposition physics was solved using an ad hoc script developed in the MATLAB^®^ (v2019b) environment. The fouling model imported the geometrical grid data from CFD simulations, reproducing the same computational mesh, with each cell having identical volume and centroid coordinates. The following equations were solved individually for each volume.

The governing equations for the colloidal fouling model were taken from the model proposed by De Jaegher et al. [42]. Here, a brief description of governing equations is presented. First, a series of parameters related to the colloids is computed.

The electrophoretic mobility, $\gamma_{p}$, of the colloids can be estimated by Hückel’s equation [49]:

(9) $$\gamma_{p}=\frac{2}{3}\frac{\epsilon_{r}\epsilon_{0}\zeta }{\mu }$$

where $\epsilon_{0}$ is the vacuum permittivity (F/m), $\epsilon_{r}$ is the relative permittivity of water (–) and *ζ* is the zeta potential (V).

Assuming the sphericity of the colloids, the charge of the colloids, *σ_p_* (C), can be computed from the zeta-potential and the local salt concentration at the membrane–solution interface through Grahame’s equation [49]:

(10) $$\sigma_{p}=4\pi {a_{p}}^{2}\sqrt{8C\epsilon_{r}\epsilon_{0}RT}sinh\left(\frac{zF\zeta }{2RT}\right)$$

where *α_p_* is the diameter of colloids (m), *T* is the absolute temperature (K) and *R* is the universal gas constant (8.314 J/mol∙K).

Once *γ_p_* is computed, the diffusion coefficient of the colloids *D_p_* (m^2^/s) can be expressed from the Nernst–Einstein relation:

(11) $$D_{p}=\frac{\gamma_{p}kT}{\sigma_{p}}$$

where *k* represents the Boltzmann constant (1.38 × 10^−23^ J/K).

The transport number for the colloids is as follows:

(12) $$t_{p}=\frac{D_{p}{z_{p}}^{2}c_{p}}{D_{p}{z_{p}}^{2}c_{p}+Dz^{2}C}$$

where *c_p_* is the concentration of the colloids and *z_p_* is the dimensionless charge of the colloids, which is expressed relative to the elementary electric charge (e = 1.6 × 10^−19^ C):

(13) $$z_{p}=\frac{\sigma_{p}}{e}$$

Finally, the colloidal rates toward the membrane surface, *J_p_*, can be expressed as follows:

(14) $$J_{p}=\frac{i\cdot t_{p}}{\sigma_{p}}$$

where *t_p_* is the colloidal transport number, which is the fraction of the current carried by the charge of the colloids.

The colloidal deposition rate on an IEM, *dm_p_*(*t*)*/dt*, is then evaluated as follows:

(15) $$\frac{dm_{p}(t)}{dt}=\alpha J_{p}$$

where *m_p_*(*t*) is the number of colloids attached to the membrane surface and α is the growth efficiency, which describes the probability of colloid deposition. Equation (14) computes the flux of colloids heading toward the membranes; however, not all the collisions occurring between colloids and membranes, or between colloids in solution and previously attached matter, lead to effective colloid deposition [50,51]. The definition and evaluation of α have been a critical aspect in the attempts to develop colloidal fouling models. In previous studies [37,38] on RO and NF systems, α was calculated using specific equations that linked its value to the potential energy barrier arising from membrane–colloid interaction. In this work, the insights and methodology reported by De Jaegher et al. [42], which highlighted the relation of α values to current density and electrical resistance in an ED unit, were used for the estimation of α in the model calculations.

The growth of the fouling layer can occur either from the adhesion of colloids on solid surfaces or from collisions with other colloids previously deposited. Therefore, at the initial stage, fouling deposition was limited to the volumes in contact with the membranes and to the spacer regions adjacent to them. In the present modelling approach, wall shear stress values, τ, were the key parameters used to determine the possible attachment of colloids on surfaces at the early stages of the process. Specifically, τ values were imported into the fouling model from the CFD simulations, and an attachment probability function, β, was modelled to be inversely proportional to the shear stress values (β ∝ 1/ τ): fouling deposition was excluded in regions with the highest wall shear stress (β = 0), while in cells characterized by the lowest shear stress value (β = 1) the colloidal deposition rate was defined according to Equation (15). Thus, the actual net colloidal deposition rate for a cell in contact with a solid surface, i.e., membranes or spacer filaments, can be expressed as follows:

(16) $$\frac{dm_{p}(t)}{dt}=\alpha {\beta J}_{p}$$

In the simulations, the model also computed the level of occupation of each cell (as the ratio between the volume of deposited colloids and the total volume of the cell). Once a cell became fully saturated by fouling, the excess colloidal deposition calculated in a timestep was transferred to neighbouring cells—*fouling spreading*. Consequently, these new cells would become new sites for colloid layer growth, as the fouling layer continued to expand.

### 2.3. Coupling CFD and Fouling Models

Fouling processes evolve over days or months, altering fluid flow and salt transport in the channels. An accurate description of these phenomena thus requires the resolution of different characteristic timescales. Specifically, fouling may require time discretization on the order of minutes, hours or days, whereas the characterization of fluid flow and mass transfer in the channels through CFD needs timesteps of seconds or fractions of a second [52]. To effectively solve both phenomena, a simulation platform was built. Specifically, CFD and fouling models were coupled through data exchange between models as follows:

First, steady-state CFD simulations were performed to solve the fluid flow and salt transport in the clean channel.

The colloidal fouling model was employed. Salt concentration distribution and wall shear stresses computed from CFD simulations were used as the input in Equations (10) and (12), and to determine whether colloids could attach to solid surfaces (membranes or spacers), respectively.

The colloid deposition rate, calculated from Equations (15) and (16), was determined from the growth efficiency α, depending, in turn, on the current density and stack resistance. Fouling simulations were carried out for a timespan on the order of hours.

After fouling deposition, CFD simulations were performed again. In this case, to account for the presence of fouling, a pseudo-viscosity matrix was calculated (representing the link between the CFD and fouling models). Specifically, the dynamic viscosity was varied from 8.9 × 10^−4^ Pa∙s (pure water) to a maximum value of 1 Pa∙s. The maximum viscosity value was imposed on the cells whose volume was completely saturated by fouling, while it changed linearly with the level of occupation of each cell. Due to the new viscosity matrix, the flow field, salt concentration spatial distribution and wall shear stresses varied with respect to the clean channel and the previous fouled condition. After the CFD simulation, updated concentration and shear stress values were imported into the fouling model.

This loop proceeded until the total experimental time duration was achieved.

**Figure 2:**
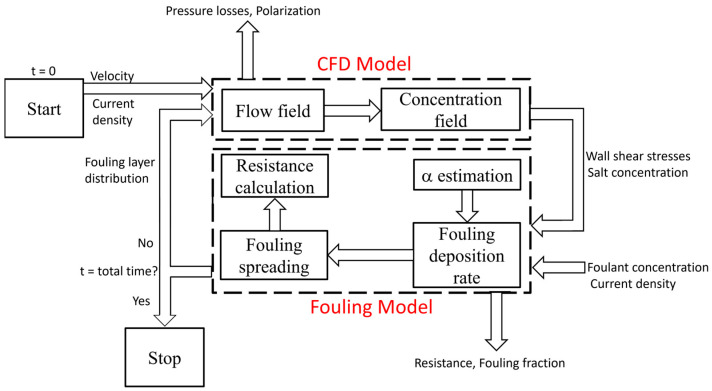
Scheme of the calculation algorithm adopted within the simulation platform.

Figure 2 is a schematic representation of the coupled model.

Each run of the colloidal fouling model covered a 6 h period, and the simulation was time-discretized into 360 equal timesteps (1 min each). For both cases, it was verified that only marginal variations in the initial CFD data and calculation accuracy would occur with a finer time discretization. Note that the assumption of a steady-state and laminar regime may appear unrealistic with a fouling layer growing inside the channel. Indeed, the reduced porosity, due to the free volume regions occupied by fouling, is expected to dictate higher flow velocities, and the higher values of dynamic viscosity, imposed in regions with fouling, may induce significant momentum gradients. The combination of these two effects can alter the flow conditions with respect to the clean channel conditions.

On the other hand, it must be noted that, although unsteady flow may be promoted from the irregular structures of the fouling layer, the characteristic timescales of flow perturbations (at most, seconds) are negligible with respect to those of fouling phenomena (at least hours). Moreover, previous studies, relative to RO systems, maintained the Newtonian fluid steady-state flow assumption even in the cases of the higher flow velocity, larger fouling extent (higher channel occupation) and larger momentum gradients (higher imposed viscosity values) of those here analysed [52,53,54].

### 2.4. Investigated Conditions

The ED experimental tests considered for model calibration and validation (Guo et al. [43]) investigated colloidal fouling phenomena in the dilute channel caused by anionic polyacrylamide (APAM), a negatively charged foulant species. Accordingly, only the dilute channel was simulated, and only the anion membrane was considered as the surface for colloid deposition. Table 1 reports the operating conditions of the experimental tests analysed by the model.

For the sake of brevity, the parameters required to solve Equations (9)–(16) are not reported here. Data can be found in Guo et al. [43].

The flow velocity inside the channel was equal to 3.6 cm/s for all the cases simulated.

### 2.5. Model Validation

A calibration procedure, described in Appendix A.1, was implemented to define a specific fouling resistance parameter, φ, linking the experimental stack resistance increase to the fouling fraction, i.e., the fraction of the total channel free volume occupied by colloids, predicted by the model. The two experimental ED tests reported by Guo et al. [43] where fouling phenomena occurred to the minimum and maximum extent—cases at a current density of 20 A/m^2^ and an APAM concentration of 0.1 g/L, and at a current density of 30 A/m^2^ and an APAM concentration of 0.3 g/L, respectively—were considered for the calibration.

The model performance in evaluating the resistance increase under different operating conditions was then tested.

**Figure 3:**
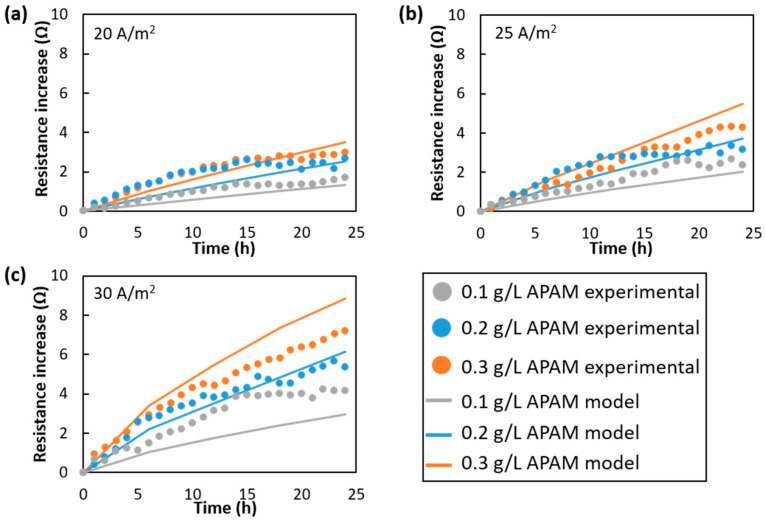
Experimental and numerical resistance increase at APAM concentrations of 0.1, 0.2 and 0.3 g/L and current densities of (**a**) 20 A/m^2^, (**b**) 25 A/m^2^ and (**c**) 30 A/m^2^. The average flow velocity was 3.6 cm/s in all tests.

Figure 3 shows experimental and numerical resistance increase profiles obtained at APAM concentrations of 0.1, 0.2 and 0.3 g/L and current densities of 20, 25 and 30 A/m^2^. In all cases, the average flow velocity inside the channel was 3.6 cm/s.

Numerical results were in very good agreement with the experimental values at current densities of 20 and 25 A/m^2^, Figure 3a,b, while at 30 A/m^2^ slightly larger discrepancies were observed for the APAM concentrations of 0.1 g/L and 0.3 g/L. Both experimental data and model predictions consistently show that the rate of resistance growth intensifies at higher APAM concentrations and current densities. This is due to the enhanced colloid flux toward the membranes driven by the current, combined with the higher availability of foulants. At a fixed current density, numerical profiles of the increasing resistance showed marked differences at varying APAM concentrations, whereas the experimental profiles appeared more closely aligned. Across all tests, resistance rose faster at the early stages of the experiments, i.e., in the first 12 h, while the fouling growth rate decreased significantly in the second half of the experiments.

Overall, the magnitude of fouling phenomena was predicted very well by the model, indicating its reliability for the detailed investigation of colloidal fouling phenomena and their effects.

## 3. Results

The validated model can provide a description of fouling phenomena at different scales. The results for both clean and fouled channels presented in this section illustrate the transition from a non-fouled unit to an ED channel affected by colloidal deposition, in terms of the fouling fraction, pressure drop and concentration polarization.

### 3.1. Flow Field and Mass Transport for the Clean Channel

**Figure 4:**
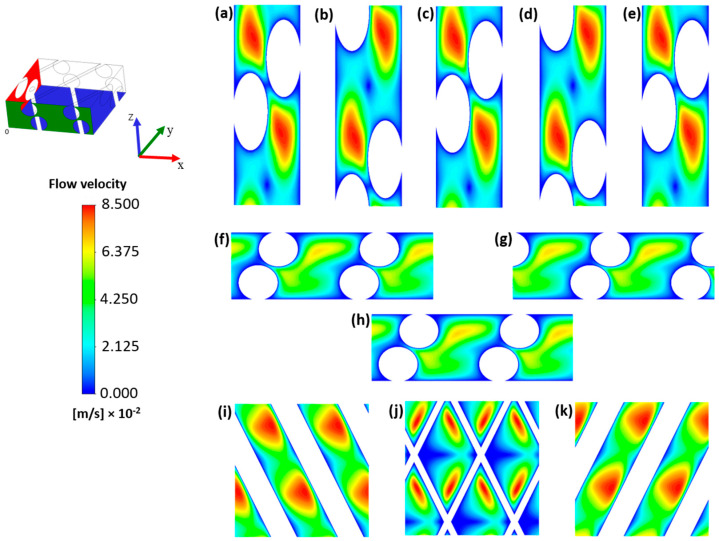
Contour plots of the flow velocity for y–x sections at x = 0 (**a**), 0.00075 m (**b**), 0.0015 m (**c**), 0.00225 m (**d**) and 0.003 m (**e**), x–z sections at y = 0 m (**f**), 0.0015 m (**g**) 0.003 m (**h**), and x–y sections at z = 0.00025 m (**i**), 0.0005 m (**j**) and 0.00075 m (**k**).

In this section, the numerical solution calculated for the clean channel, prior to the onset of colloidal fouling, is analysed. This serves as a reference for the CFD simulations with an integrated fouling layer. Figure 4 shows contour plots of the flow velocity at several slices of the clean channel.

Due to the Unit Cell approach and the imposed periodic boundary conditions on lateral walls, i.e., the walls having either the x- or y-axis as their normal, the flow pattern was completely established from the plane located at x = 0 m. The periodic boundary conditions also ensured regular flow patterns repeating along the x-direction; see Figure 4a–e. Identical contour plots can be seen at x = 0 m, x = 0.0015 m and x = 0.003 m, and at x = 0.00075 and x = 0.00225. This is attributed to the regularity of the spacer and the two spacer meshes considered. For the same reason, repeating regular patterns were also found along the y-direction, with the calculated solution coinciding at y = 0 and y = 0.003 m; see Figure 4f,h. The presence of the spacer disrupted the typical parabolic velocity profile characteristic of laminar flow inside a rectangular cross-sectional duct [55]. A more complex flow regime was instead observed, with regions of high velocity (a maximum of 0.085 m/s with 0.036 m/s as average flow velocity) distributed across the domain, reducing the extent of near-stagnant zones adjacent to the spacer filaments and membrane surfaces. Although the main velocity and pressure gradient were imposed only along the x-axis, the tortuous path induced by the spacer resulted in notable velocity components also along the y- and z-axes. Wider regions with low velocity were found at z = 0.0005—see Figure 4j—due to the larger area covered by the spacer filaments at this channel height. Along the z-axis, similar flow structures were observed at equal distances from the membranes, see Figure 4i,k. These flow features are consistent with previous CFD studies on spacer-filled membrane channels [56,57,58].

**Figure 5:**
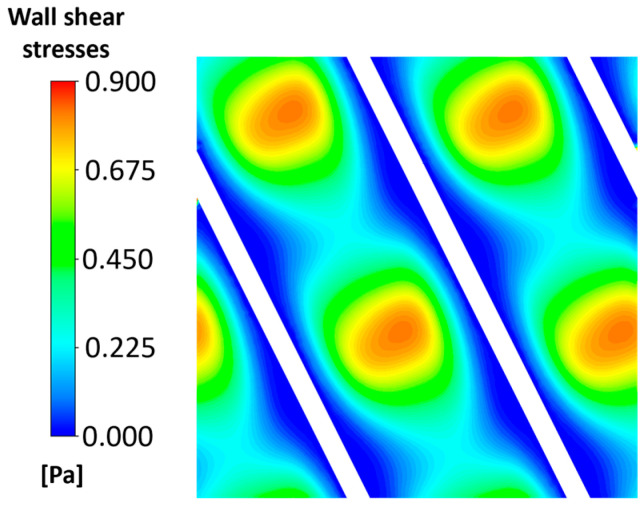
Contour plot of wall shear stresses for x–y section at z = 0. The average flow velocity was equal to 3.6 cm/s.

The effect of the flow field on the membrane surface can be further analysed by examining the distribution of wall shear stress, as shown in Figure 5.

The contour plot highlights alternating regions of high and low shear stress, corresponding to the flow perturbations induced by the spacer. Higher-shear stress zones are mainly located in regions where local velocity gradients are more pronounced. Conversely, the blue regions indicate low-shear stress areas, typically occurring in the recirculation or stagnant flow regions between filaments and membranes [59,60]. This heterogeneous shear stress pattern plays a key role in controlling mass transfer efficiency and fouling behaviour at the membrane interface [61,62].

**Figure 6:**
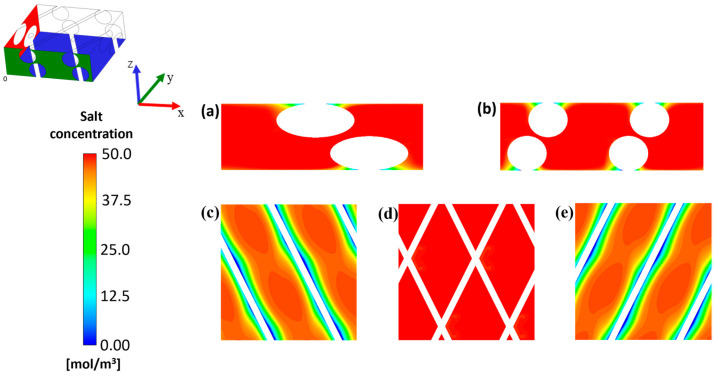
Contour plots of the salt concentration at x = 0.0015 m (**a**) and y = 0.0015 m (**b**), and at z = 0 m (**c**), 0.0005 m (**d**) and 0.001 m (**e**), for a current density of 30 A/m^2^.

The effect of shear stresses and flow velocity on concentration profiles is also shown in Figure 6.

The presence of the spacer guaranteed a very good concentration uniformity. The salt concentration remained nearly constant along the z-direction; see Figure 6a,b. Low concentration values can be observed only in regions where the spacer is almost in contact with the membrane, see Figure 6c,d. This is due to the scarce local fluid dynamic conditions (*shadow regions*), as highlighted by the wall shear stress distribution in Figure 5, that lead to low mass transfer performance [62,63]. Limited polarization phenomena were detected inside the channel, even considering the highest current density among the cases considered, i.e., 30 A/m^2^, which represents the most challenging condition due to the highest salt output flux. The reduction in polarization coefficients with current density is, indeed, attributed to the higher salt outflow from the channel. This is typical and largely consistent with studies in the literature [29,64,65].

For the clean channel, the θ values were 0.84, 0.80 and 0.77 for the current densities of 20 A/m^2^, 25 A/m^2^ and 30 A/m^2^, respectively.

### 3.2. Fouled Channel

**Figure 7:**
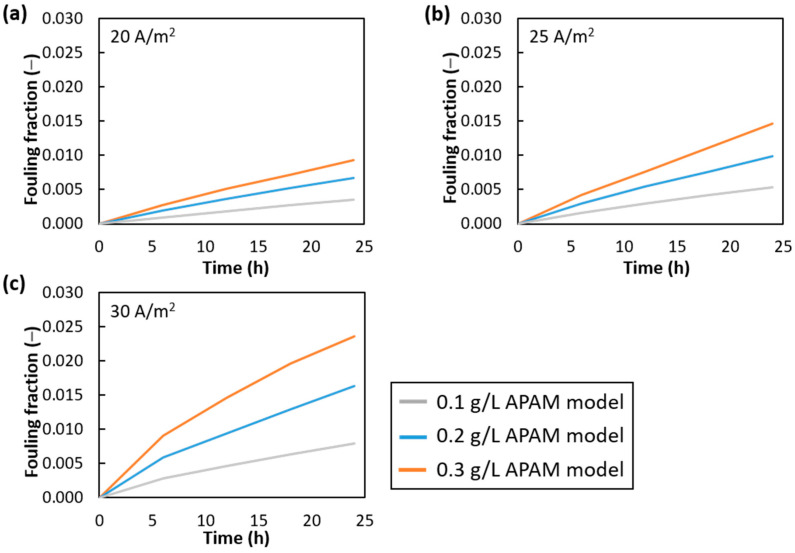
Numerical fouling fraction at APAM concentrations of 0.1, 0.2 and 0.3 g/L and current densities of (**a**) 20 A/m^2^, (**b**) 25 A/m^2^ and (**c**) 30 A/m^2^.

Figure 7 presents the fouling fraction evolution over-time at APAM concentrations of 0.1, 0.2, and 0.3 g/L and current densities of 20, 25 and 30 A/m^2^.

The steepest rise in fouling fraction occurred at the early stages of the tests (i.e., the first 6 h). This is due to the fact that the regions where the spacers are in contact with the anion membrane presented the lowest wall shear stress values and, at the same time, the lowest salt concentration values; see Figure 5 and Figure 6. The former enhanced the attachment probability function, making these the first regions where the fouling layer was formed; the latter increased the colloidal deposition rates (Equations (9)–(16)), which were highest at the beginning.

The difference between the cases at different APAM concentrations was more marked with increasing current density. The overall fouling fraction remained on the order of a few percent, with most cases showing a final value below 1%. The maximum value of ~2.5% was observed for the case at a current density of 30 A/m^2^ and an APAM concentration of 0.3 g/L, which exhibited the most pronounced fouling extent.

The formation of a fouling layer on the membrane surface or around the spacer filaments induced variations in fluid dynamics and salt transport.

**Figure 8:**
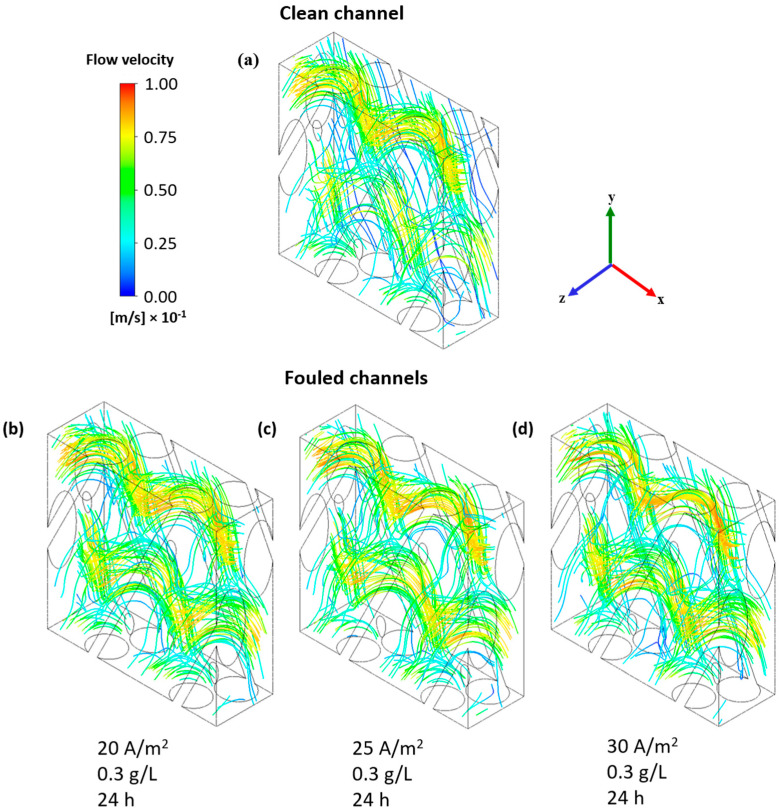
Solution velocity streamlines in (**a**) the clean channel and in the fouled channel after 24 h for the cases at an APAM concentration of 0.3 g/L and current densities of (**b**) 20 A/m^2^, (**c**) 25 A/m^2^ and (**d**) 30 A/m^2^.

Figure 8 shows the solution velocity streamlines for four cases: the clean channel and the fouled channel after 24 h for an APAM concentration of 0.3 g/L at current densities of 20, 25 and 30 A/m^2^.

The velocity increased from the clean to the fouled channels, with a slight enhancement at higher current densities, as evidenced by the more reddish zones in Figure 8. A more complex flow field is depicted by the streamlines in fouled channels; for instance, the regular blue lines in Figure 8a located near the membrane at z = 0, i.e., the AEM surface, disappeared in Figure 8b–d, suggesting flow alterations induced by the growth of the fouling layer on the membrane. These flow alterations were more intense with increasing current density, since more extensive colloidal deposition phenomena occurred, as shown in Figure 4.

**Figure 9:**
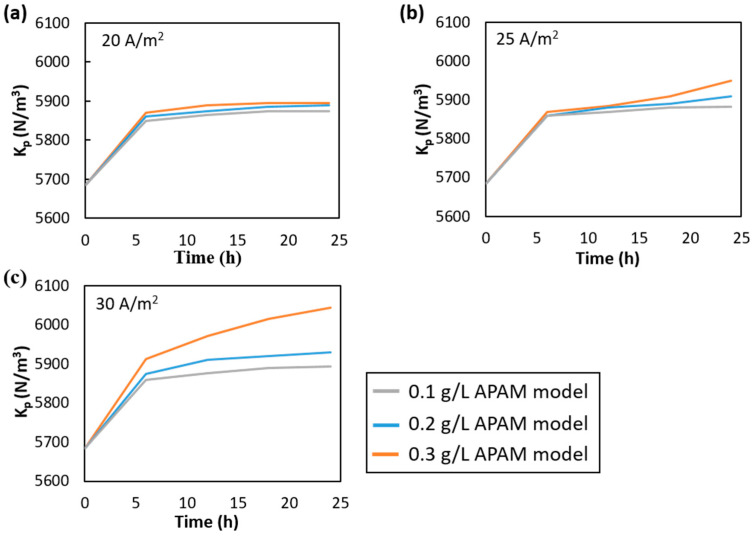
Numerical pressure gradient *K_p_* at APAM concentrations of 0.1, 0.2 and 0.3 g/L and current densities of (**a**) 20 A/m^2^, (**b**) 25 A/m^2^ and (**c**) 30 A/m^2^.

Figure 9 shows the imposed pressure gradients, *K_p_*, throughout the duration of the tests. The pressure gradient along the x-axis, corresponding to the main flow direction, determined the flow velocity in the channel, and its value was adjusted to maintain an average flow velocity of 3.6 cm/s.

**Figure 10:**
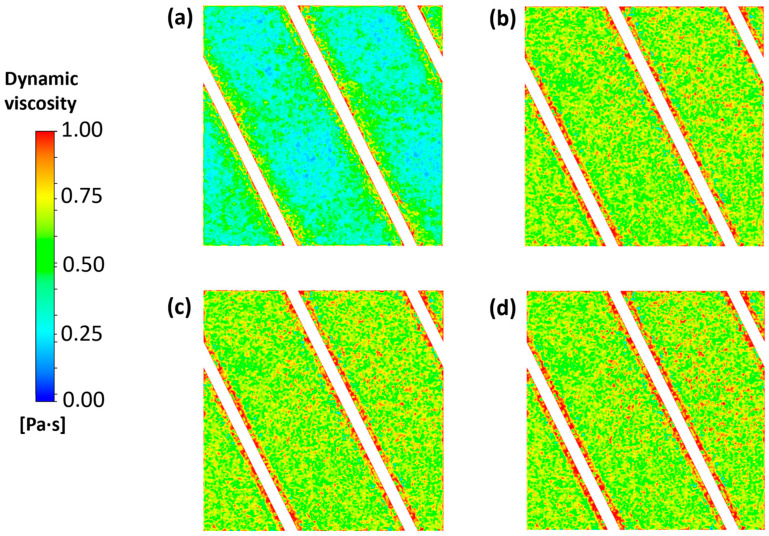
Dynamic viscosity at the AEM surface for the case at a current density of 20 A/m^2^ and an APAM concentration of 0.3 g/L after (**a**) 6 h, (**b**) 12 h, (**c**) 18 h and (**d**) 24 h.

From Figure 9, the pressure gradient increased over time, indicating that the fouling layer resulted in a higher flow resistance. Consequently, greater pumping power would be required to maintain the same flow velocity in the presence of fouling, leading to higher operational costs. For all cases, the steepest and most pronounced increase was observed during the first run of the simulation platform, with an almost plateau-like behaviour detected after the first 10 h in most instances. This is attributed to the fact that the earliest deposited colloids filled the intersections between the anion membrane and the spacer, causing the most significant changes in channel geometry; see Figure 10. Once the initial colloidal deposition occurred, the subsequent fouling layer growth mainly restricted the channel dimension. At a fixed current density, very similar trends and values were observed at current densities of 20 and 25 A/m^2^, whereas a marked difference was found at 30 A/m^2^ across the three APAM concentrations. For APAM concentrations of 0.2 and 0.3 g/L and for current densities of 25 and 30 A/m^2^, *Kp* continued to increase during the last 6 h. The pressure gradient, and hence the associated pressure losses, increased by approximately 5–10% compared to the initial value for the clean channel.

For the most severe case, i.e., 30 A/m^2^ and 0.3 g/L of APAM, the pressure gradient varied from ~5700 to ~6050 N/m^3^.

A close inspection of regions affected by fouling layer formation is shown in Figure 10, which presents contour plots of the dynamic viscosity over the AEM at different times for the case at a current density of 20 A/m^2^ and an APAM concentration of 0.3 g/L. As explained in Section 2.3, viscosity values are related to the degree of local fouling, thus providing an indication of the level of occupation of colloid occupation in the regions in contact with the membranes.

Figure 10 clearly highlights that the areas most susceptible to fouling are those near the contact points between the spacer filaments and the membranes, due to the previously discussed effects of velocity and concentration (dead zones). These observations align with experimental evidence documented in the literature [17,53]. The fouling layer was not uniform, which can be attributed to the complex wall shear stress distribution induced by the spacer, see Figure 5.

The fouling layer initially deposited mainly on membrane surface regions near the spacer, as seen in Figure 10a. However, at later stages, even the membrane areas not in direct contact with the spacer became covered by a fouling layer; see Figure 10b–d.

**Figure 11:**
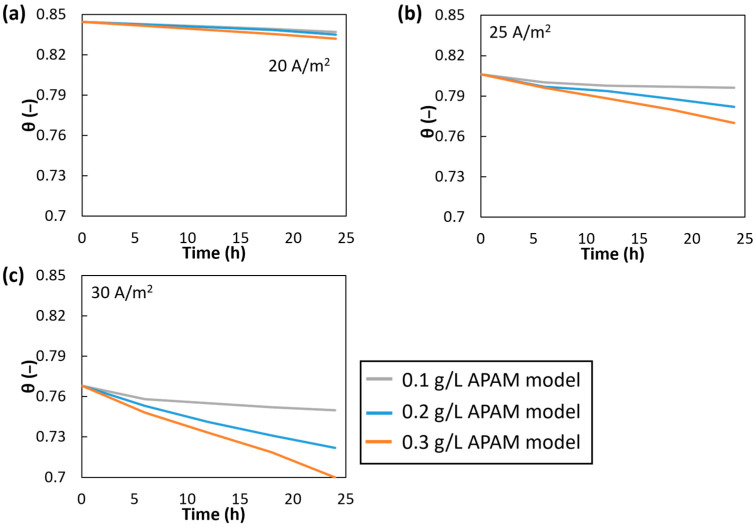
Numerical polarization coefficients θ at APAM concentrations of 0.1, 0.2 and 0.3 g/L and current densities of (**a**) 20 A/m^2^, (**b**) 25 A/m^2^ and (**c**) 30 A/m^2^.

The establishment of the fouling layer affected not only the pressure losses but also the salt concentration. Consequently, the average salt concentration at the membrane surfaces evolved over time. Figure 11 shows the concentration polarization coefficients for all cases investigated. Note that the $\hat{C_{w}}$ values in Equation (8) refer here only to the anion membrane surface (the main membrane where fouling occurs).

A more rapid reduction in polarization effects was observed with increasing current density and higher APAM concentrations. The strongest reduction in polarization coefficients occurred for the cases at 30 A/m^2^ and APAM concentrations of 0.2 and 0.3 g/L, the final polarization coefficients being 0.72 and 0.70, respectively. In all the cases at an APAM concentration of 0.1 g/L, the polarization coefficients showed a significant and rapid decline during the first run of the model, followed by an almost plateaued behaviour for the remaining test duration. Conversely, all cases at an APAM concentration of 0.3 g/L exhibited a continuous reduction over time. An intermediate behaviour was observed at an APAM concentration of 0.2 g/L, showing a more pronounced decrease at higher current densities; in particular, the case at 30 A/m^2^ displayed a continuous decline over time.

Apart from the two cases most affected by fouling, the decrease in polarization coefficients over 24 h was consistently less than 10% and in some instances, particularly at 20 A/m^2^, below 5%.

## 4. Conclusions

In this work, an original simulation platform was developed to create a comprehensive modelling tool for colloidal fouling prediction in electrodialysis units. Results from studies in the literature were exploited to refine and calibrate the overall model. The coupled model demonstrated the ability to simulate and quantify the magnitude of colloidal deposition under varying operating conditions, as confirmed by comparisons with experimental data. CFD simulations were performed to investigate the effect of fouling, from a fluid dynamics standpoint, within an ED feed channel equipped with a spacer. The analysis assessed the influence of current density on polarization phenomena in both clean and fouled channels, highlighting how polarization coefficients decrease with increasing current density and fouling layer thickness.

Severe fouling phenomena were observed at the highest current density, as evidenced by the polarization coefficients and pressure loss analysis, whereas minimal fouling occurred at the lowest current density. More complex flow trajectories were observed in fouled channels compared to clean ones, due to the portion of the channel occupied by fouling and the structures formed around the spacer. The results also indicated that the regions most susceptible to fouling were those with the lowest local concentration and flow velocity. Specifically, the fouling layer initiated near the contact points between the spacers and the membrane surface, before spreading across the membrane surface.

In conclusion, the presented model provides a valuable tool for investigating the effects of colloidal fouling at both macroscopic (resistance and pressure losses) and microscopic (polarization, concentration distribution, and fouling fraction) scales. It is therefore a practical tool for predicting fouling phenomena and for guiding control and mitigation strategies in electrodialysis operations.

## Figures and Tables

**Table 1:** Experimental conditions from electrodialysis tests performed by Guo et al. [43].

Experimental Conditions	
Feed solution concentration	0.05 mol/L NaCl + APAM
APAM concentration	0.1–0.2–0.3 g/L
Current density	20–25–30 A/m^2^
Total test time	24 h

**Figure A1:**
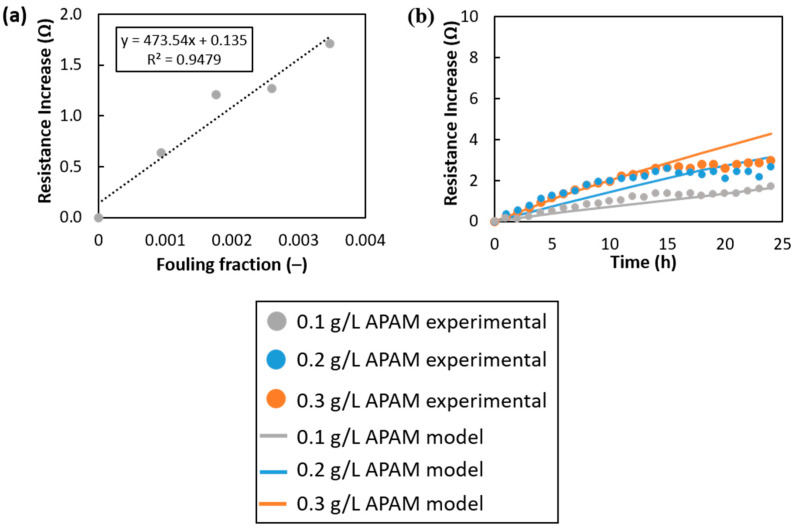
(**a**) Calibration between resistance increase and fouling fraction at current density of 20 A/m^2^ and APAM concentration of 0.1 g/L. (**b**) Numerical and experimental resistance increase profiles at current density of 20 A/m^2^ and APAM concentration of 0.1, 0.2 and 0.3 g/L.

**Figure A2:**
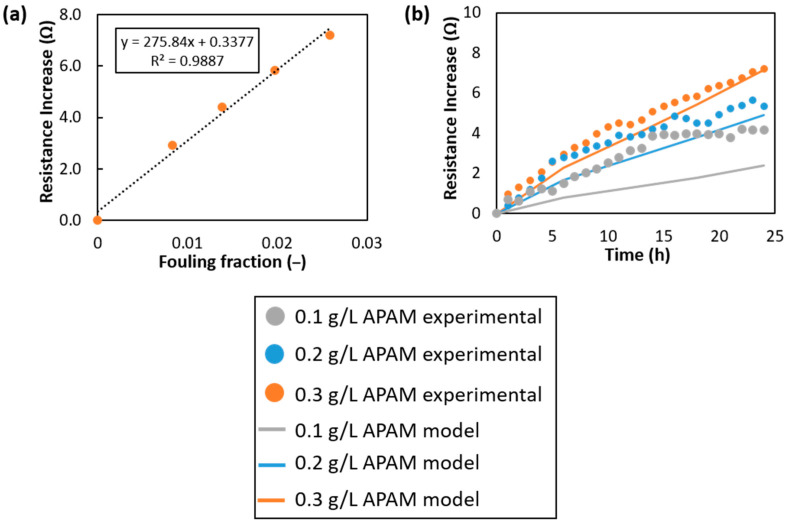
(**a**) Calibration between resistance increase and fouling fraction at current density of 30 A/m^2^ and APAM concentration of 0.3 g/L. (**b**) Numerical and experimental resistance increase profiles at current density of 30 A/m^2^ and APAM concentration of 0.1, 0.2 and 0.3 g/L.

**Figure A3:**
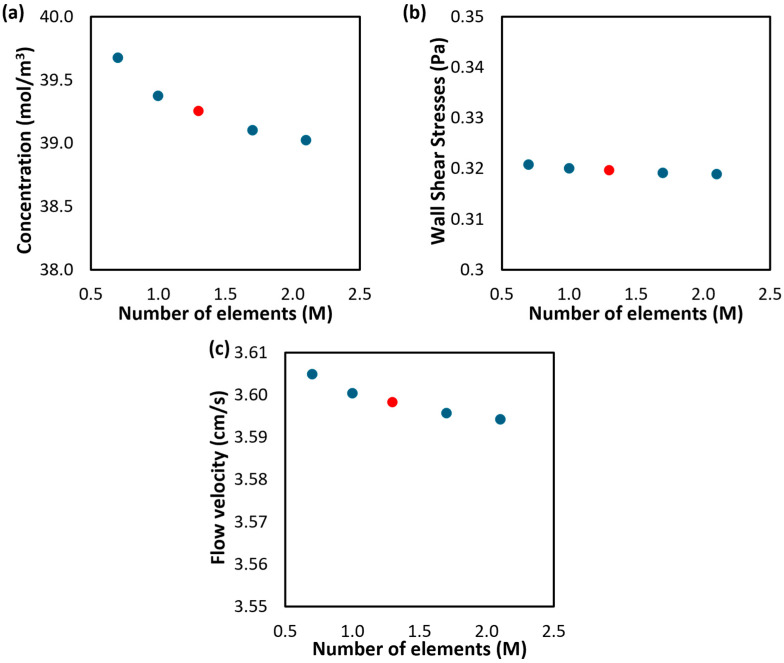
Grid-dependence study. (**a**) Area-averaged salt concentration and (**b**) area-averaged wall shear stress calculated at the AEM surface, and (**c**) volume-averaged flow velocity, at a current density of 30 A/m^2^ for the clean channel using different grids. The red dot in each graph corresponds to the grid adopted in this work to perform the simulations whose results are reported in the main text.

## Data Availability

Data will be made available on request.

## Nomenclature

A	Membrane surface area in a Unit Cell (m^2^)
$a_{p}$	Colloid diameter (m)
C	Molar salt concentration (mol/m^3^)
$C_{b}$	Molar bulk salt concentration (mol/m^3^)
$\hat{C_{b}}$	Mean molar bulk salt concentration (mol/m^3^)
C_0_	Molar solvent concentration (mol/m^3^)
$c_{p}$	Colloid concentration (mol/m^3^)
$C_{w}$	Molar salt concentration at membrane–solution interface (mol/m^3^)
$\bar{C_{w}}$	Mean molar salt concentration at membrane–solution interface (mol/m^3^)
D	Salt diffusion coefficient (m^2^/s)
$D_{p}$	Colloid diffusion coefficient (m^2^/s)
F	Faraday constant (C/mol)
**i**	Current density (A/m^2^)
J	Salt flux (mol/m^2^∙s)
$J_{p}$	Colloid flux towards membrane (1/m^2^∙s)
k	Unit vector of main flow direction
$K_{C}$	Concentration gradient along the main flow direction (mol/m^4^)
$K_{p}$	Pressure gradient along the main flow direction (N/m^3^)
$m_{p}$	Number of APAM colloids attached to the membrane (–)
p	Pressure (Pa)
R	Universal gas constant (J/mol∙K)
T	Temperature (K)
t	Transport number (–)
$t_{p}$	Colloid transport number (–)
**u**	Velocity vector (m/s)
$u_{0}$	Solvent velocity vector (m/s)
$u_{main}$	Velocity along main direction (m/s)
$u_{main\;ave}$	Mean velocity along main direction (m/s)
z	Valence number (–)
$z_{p}$	Colloid dimensionless charge (–)
**Greek**	
α	Growth efficiency (–)
β	Attachment probability (–)
$\gamma_{p}$	Colloid electrophoretic mobility (m^2^/s∙V)
Δp	Pressure losses (Pa)
$\epsilon_{0}$	Vacuum permittivity (F/m)
$\epsilon_{r}$	Relative permittivity of water (–)
ζ	Zeta potential (V)
η	Dynamic viscosity of solution (Pa∙s)
θ	Polarization coefficient
$k_{T}$	Boltzmann constant (J/K)
ρ	Density (kg/m^3^)
$\sigma_{p}$	Colloid charge (C)
τ	Wall shear stress (Pa)
φ	Specific fouling resistance (Ω/fouling fraction)
**Abbreviations**	
APAM	Anion polyacrylamide
AEM	Anion-exchange membrane
CEM	Cation-exchange membrane
IEM	Ion-exchange membrane
ED	Electrodialysis

## Appendix A

### Appendix A.1. Model Calibration

The model was calibrated against the literature data to correlate the degree of fouling fraction predicted by the model with the experimental electrical resistance increase. In the calibration, the coupled model was employed as described in Section 2.4 to simulate the experimental cases reported in Table 1, but only experimental resistance values were taken into account to estimate α values for the fouling simulations. At the end of simulation loops (CFD-fouling coupled simulations), a linear regression was performed between experimental resistance increase values and fouling fraction values predicted by the model at the same corresponding times. The slope of the best-fitting straight line was taken as the calibration parameter linking the numerical fouling fraction to the ED unit resistance, i.e., the specific fouling resistance φ.

As reported in the main text, the two experiments from Guo et al. [43], characterized by the least and the most severe fouling conditions, were selected for model calibration: (i) the case with a current density of 20 A/m^2^ and an APAM concentration of 0.1 g/L, i.e., the lowest current density and lowest APAM concentration; (ii) the case with a current density of 30 A/m^2^ and an APAM concentration of 0.3 g/L, i.e., the highest current density and highest APAM concentration. At this point in model testing, only the initial ED unit resistance was adopted to estimate α for the first fouling simulation, whereas for subsequent simulations, the resistance increase was estimated from the numerical fouling fraction through φ.

Figure A1a presents the specific resistance value calculated for the test at a current density of 20 A/m^2^ and an APAM concentration of 0.1 g/L. Additionally, Figure A1b shows the numerical resistance profiles calculated using this specific resistance value.

A value of 473.54 Ω/fouling fraction yielded the best agreement for the case least affected by fouling. When this value was applied to all the cases at the same current density, the numerical results showed very good agreement with experimental data, which also extended to the case not included in the calibration phase.

Figure A2a reports the value of the specific resistance calculated for the test at a current density of 30 A/m^2^ and an APAM concentration of 0.3 g/L. Moreover, Figure A2b shows the numerical resistance profiles for the cases at the same current density, calculated with this specific resistance value.

A value of 275.84 Ω/fouling fraction was found to provide the best agreement for the case most affected by colloidal fouling phenomena. A good agreement between the model and the experimental data was found for the cases at the same current density, with a slight underestimation for the case at the lowest APAM concentration.

Finally, the average φ value of 374.7 Ω/fouling fraction from the two calibration cases was used for all the cases reported in the main text.

### Appendix A.2. Grid-Dependence Study

Hybrid grids combining hexahedral and tetrahedral elements were adopted. The use of hybrid meshes was necessary due to the complex shape of the spacer filaments, which could not be adequately discretized with purely hexahedral elements. However, the overall meshes remained predominantly hexahedral.

A grid-dependence analysis was performed. Both local and global quantities were compared as functions of the total number of volumes, considering a clean channel at the highest current density case of 30 A/m^2^. Five grids were considered for this study. Figure A3 reports the area-averaged salt concentration and area-averaged wall shear stresses at the AEM surface, and the volume-averaged flow velocity. Note that, the velocity slightly changed since the same pressure gradient was imposed in each simulation.

From Figure A3, it can be observed that the mesh adopted in this work (1.3 M elements) showed negligible maximum deviation (lower than 2%) compared with the finest grid used in the grid-dependence study (2.1 M elements), thereby confirming that the numerical results are effectively independent of the discretization level.

For the adopted grid, the average characteristic size was equal to 2 × 10^−5^ m.

The average element quality and orthogonal quality were 0.84 (with a standard deviation of 0.1192) and 0.80 (with a standard deviation of 0.114), respectively. These metrics demonstrate that the generated mesh achieves a very good overall quality, according to the mesh quality benchmarks provided in previous studies [66,67,68].

A refinement consisting of eight layers with a thickness of ~5 μm was added to properly resolve the regions near the membrane surfaces and spacer filaments.
